# Supplementary material for: Targeting of Ubiquitin E3 Ligase RNF5 as a Novel Therapeutic Strategy in Neuroectodermal Tumors
Source: Cancers (Basel). 2022 Apr 1;14(7):1802. doi: 10.3390/cancers14071802 (PMC8997491; doi:10.3390/cancers14071802)
Supplement: Supplementary file 1 [file cancers-14-01802-s001.zip › cancers-1645907-supplementary.pdf]

cancers

MDPI

Article

Targeting of Ubiquitin E3 Ligase RNF5 as a Novel Therapeutic Strategy in Cancer

Elisa Principi, Elvira Sondo, Giovanna Bianchi, Silvia Ravera, Martina Morini, Valeria Tomati, Cristina Pastorino, Federico Zara, Claudio Bruno, Alessandra Eva, Nicoletta Pedemonte and Lizzia Raffaghello

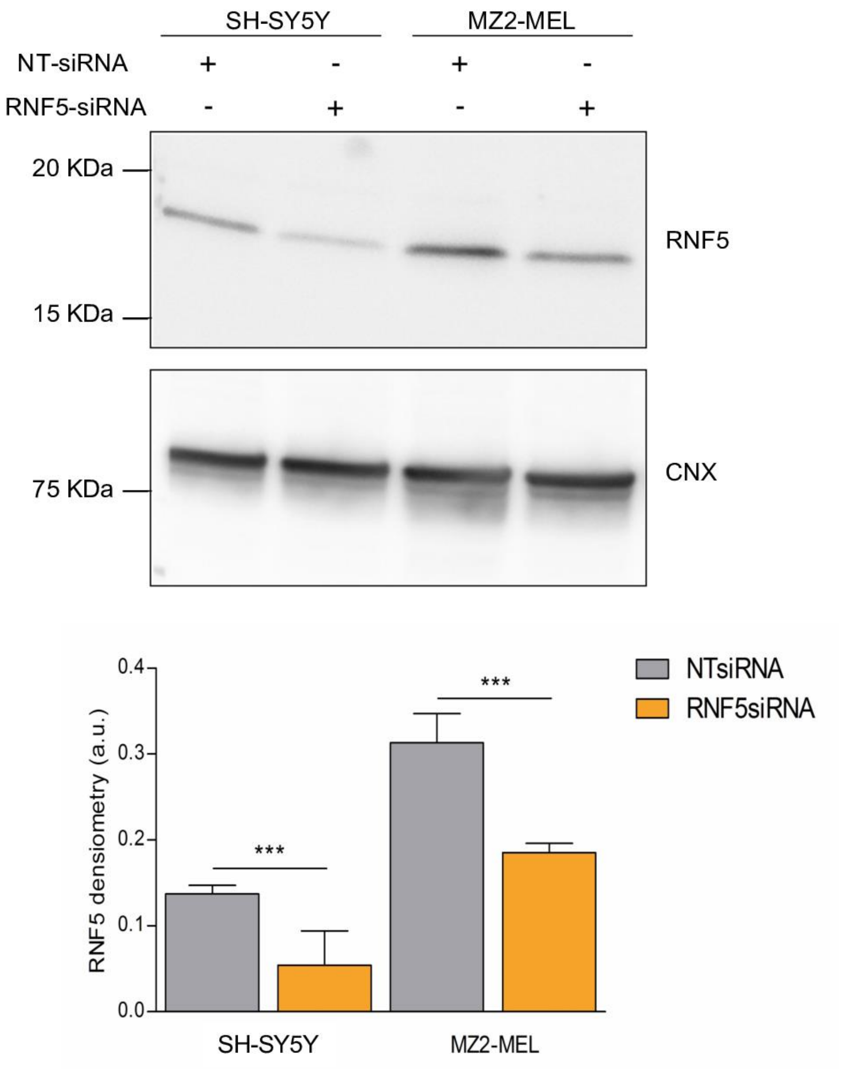

Figure S1. RNF5 expression and silencing efficiency on neuroblastoma and melanoma cell lines. Representative western blot analysis and densitometric quantification of RNF5 expression in human neuroblastoma SH-SY5Y and melanoma MZ2-MEL cell lines following transfection with Non-Targeting (NT) or RNF5 siRNA. Calnexin (CNX) is used as loading control. Results are expressed as mean ± SD from three different experiments. Statistical analysis was performed using Unpaired t Test. Asterisks indicates statistical significance of RNF5 siRNA versus NT-siRNA: ***, $p < 0.001$.

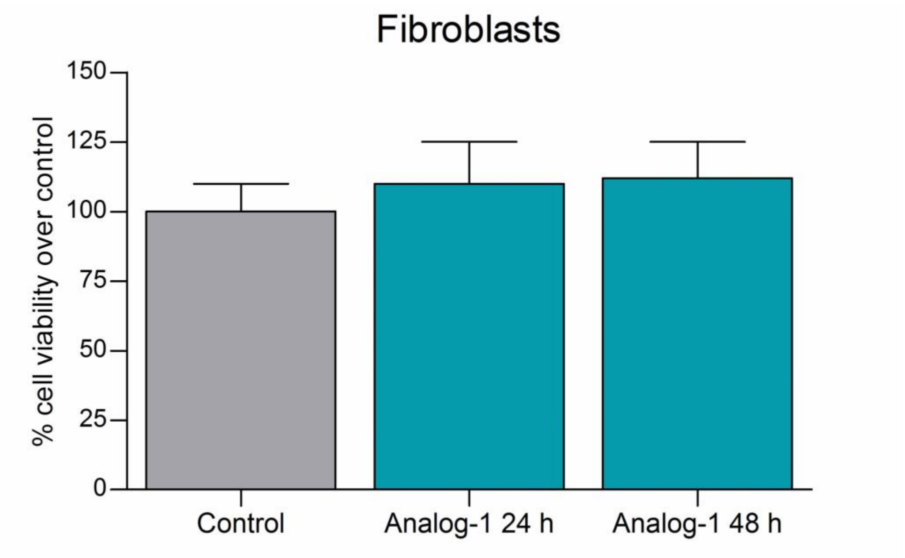

Figure S2. Analog-1 does not affect the cell viability of human fibroblasts. Cell viability of human fibroblasts treated with 10 μM Analog-1 for 24 and 48 hours was determined by Trypan Blue Assay. Results are expressed as mean of the percentage of viable cells over control ± SD from three different experiments. Statistical analysis was performed using Unpaired t Test.

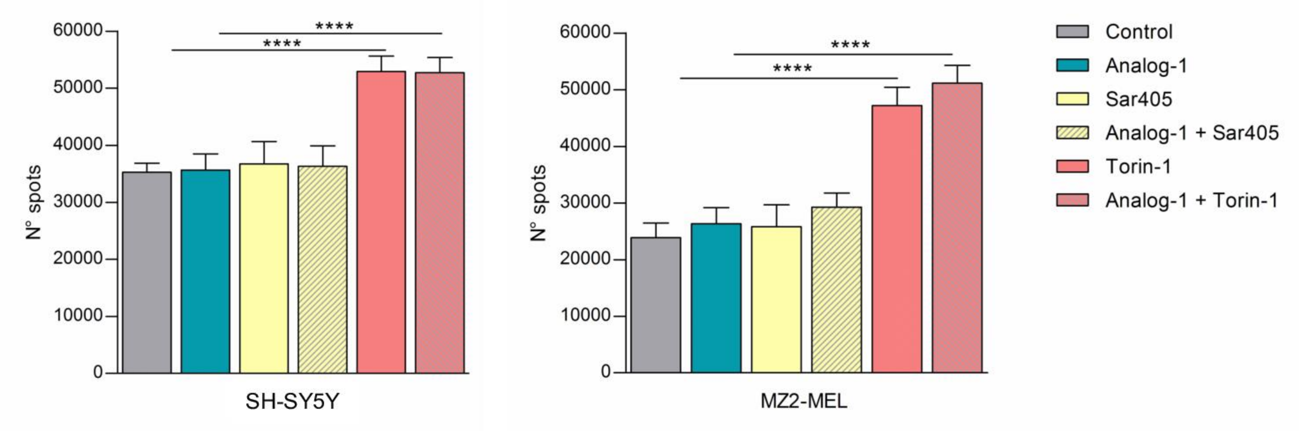

Figure S3. Analog-1 does not affect the autophagy of neuroblastoma and melanoma cells. Tumor cell autophagy was evaluated in SH-SY5Y and MZ2-MEL cells treated with vehicle alone (negative control) or Analog-1 10μM or SAR405 (autophagy inihibitor) or Torin-1 (autophagy inducer) for 24 hours. The cells were stained with 50 μM monodansylcadaverine (MDC) and imaged imaged by using the Opera Phenix high-content screening system. Data were expressed as means of n° spots ± SEM (n = 3). Statistical analysis was performed using Unpaired t Test.

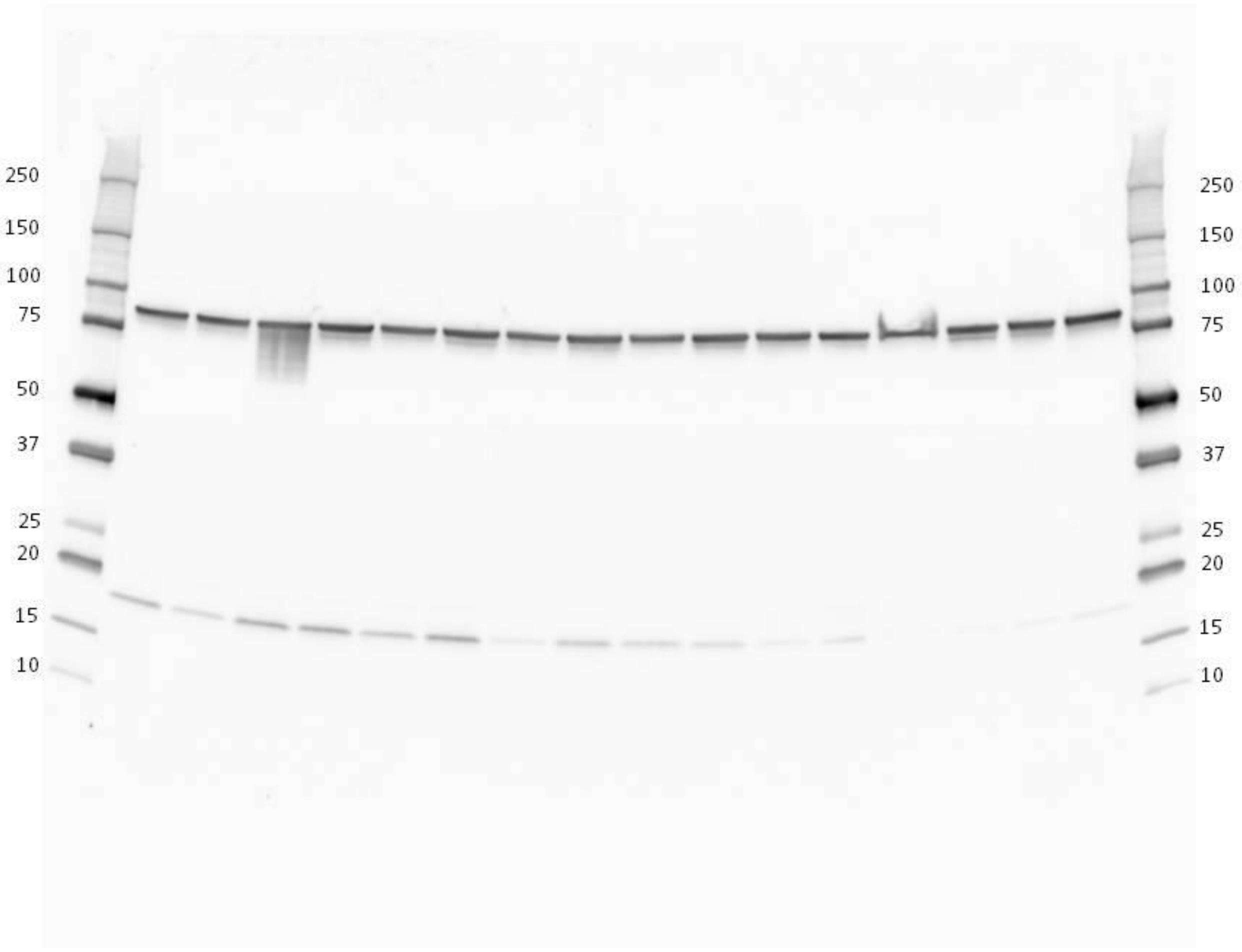

Figure S4. Figure 2A calnexin exposure.

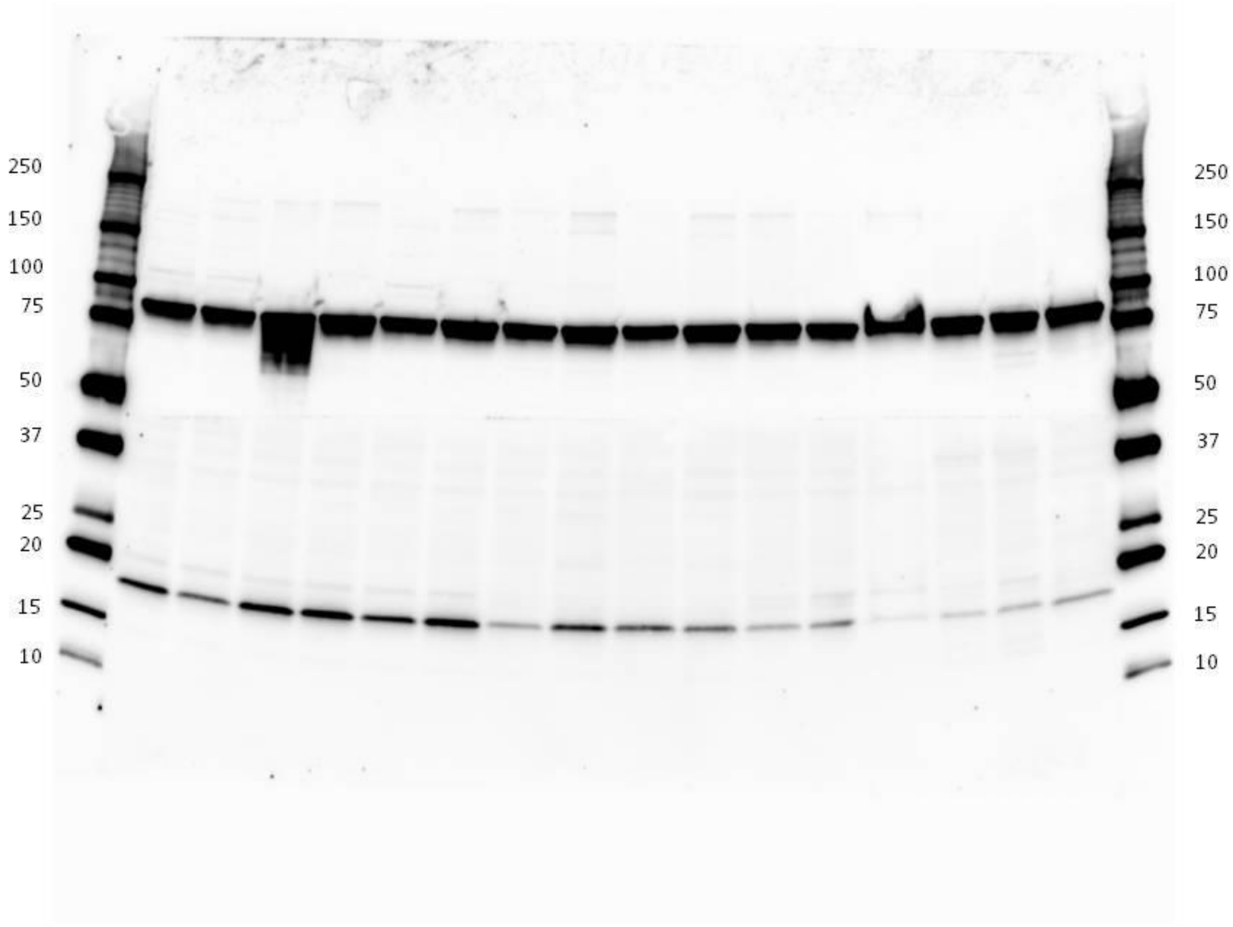

Figure S5. Figure 2A RNF5 exposure.

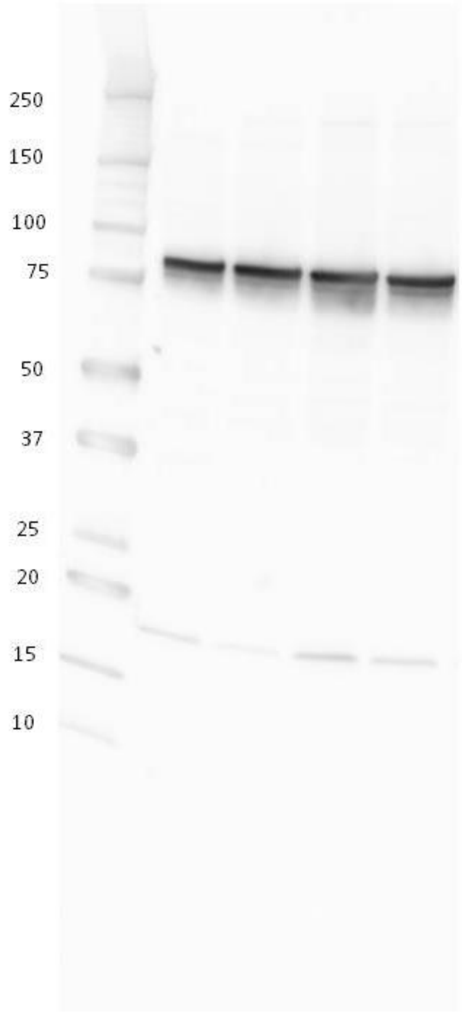

Figure S6. Figure S1 calnexin exposure.

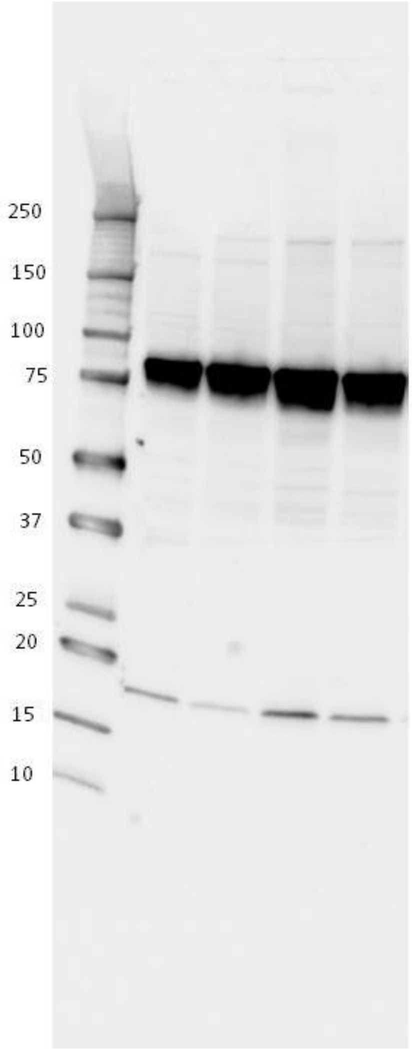

Figure S7. Figure S1 RNF5 exposure.

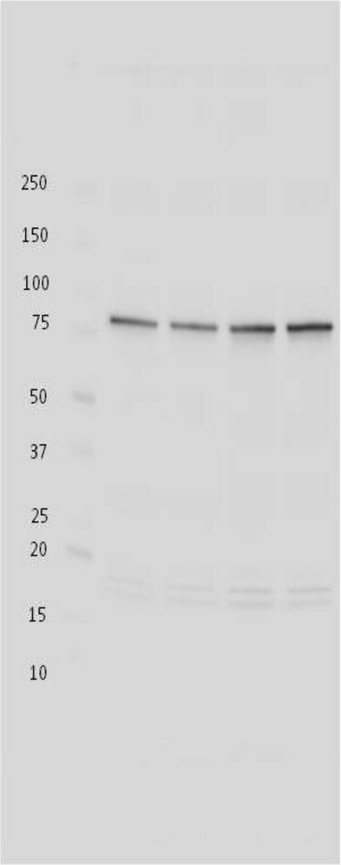

Figure S8. Figure 2D calnexin exposure.

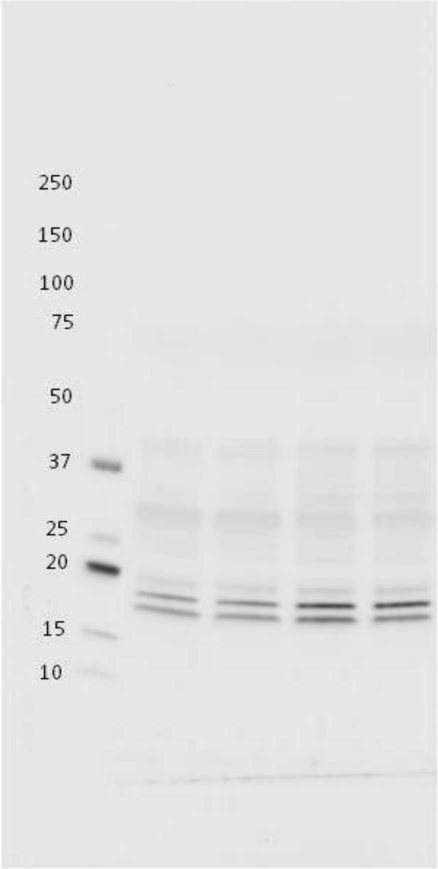

Figure S9. Figure 2D RNF5 exposure.
